# Stop or go? Preventive cognitive therapy with guided tapering of antidepressants during pregnancy: study protocol of a pragmatic multicentre non-inferiority randomized controlled trial

**DOI:** 10.1186/s12888-016-0752-6

**Published:** 2016-03-18

**Authors:** Nina M. Molenaar, Marlies E. Brouwer, Claudi L. H. Bockting, Gouke J. Bonsel, Christine N. van der Veere, Hanneke W. Torij, Witte J. G. Hoogendijk, Johannes J. Duvekot, Huibert Burger, Mijke P. Lambregtse-van den Berg

**Affiliations:** Department of Clinical Psychology, Utrecht University, Heidelberglaan 1, 3584 CS Utrecht, The Netherlands; Department of Psychiatry, Erasmus Medical Centre, ’s Gravendijkwal 230, 3015 CE Rotterdam, The Netherlands; Department of Obstetrics and Gynaecology, Erasmus Medical Centre, ’s Gravendijkwal 230, 3015 CE Rotterdam, The Netherlands; Department of Pediatrics, University Medical Centre Groningen, Hanzeplein 1, 9713 GZ Groningen, The Netherlands; Department of obstetrics and birth care, Hogeschool Rotterdam, Rochussenstraat 198, 3015 EK Rotterdam, The Netherlands; Department of General Practice, University Medical Centre Groningen, Antonius Deusinglaan 1, 9713 AV Groningen, The Netherlands; Department of Child and Adolescent Psychiatry, Erasmus Medical Centre – Sophia Childrens Hospital, Wytemaweg 80, 3015 CN Rotterdam, The Netherlands

**Keywords:** Pregnancy, Antidepressants, Selective serotonin reuptake inhibitors, SSRI, Depression, Prevention, Relapse, Recurrence, Cognitive therapy, Perinatal outcome, Cost-effectiveness

## Abstract

**Background:**

Approximately 6.2 % of women in the USA and 3.7 % of women in the UK, use Selective Serotonin Reuptake Inhibitors (SSRIs) during their pregnancies because of depression and/or anxiety. In the Netherlands, this prevalence is around 2 %. Nonetheless, SSRI use during pregnancy is still controversial. On the one hand SSRIs may be toxic to the intrauterine developing child, while on the other hand relapse or recurrence of depression during pregnancy poses risks for both mother and child. Among patients and professionals there is an urgent need for evidence from randomized studies to make rational decisions regarding continuation or tapering of SSRIs during pregnancy. At present, no such studies exist.

**Methods/Design:**

‘Stop or Go’ is a pragmatic multicentre randomized non-inferiority trial among 200 pregnant women with a gestational age of less than 16 weeks who use SSRIs without clinically relevant depressive symptoms. Women allocated to the intervention group will receive preventive cognitive therapy with gradual, guided discontinuation of SSRIs under medical management (STOP). Women in the control group will continue the use of SSRIs (GO). Primary outcome will be the (cumulative) incidence of relapse or recurrence of maternal depressive disorder (as assessed by the Structured Clinical Interview for DSM disorders) during pregnancy and up to three months postpartum. Secondary outcomes will be child outcome (neonatal outcomes and psychomotor and behavioural outcomes up to 24 months postpartum), and health-care costs. Total study duration for participants will be therefore be 30 months. We specified a non-inferiority margin of 15 % difference in relapse risk.

**Discussion:**

This study is the first to investigate the effect of guided tapering of SSRIs with preventive cognitive therapy from early pregnancy onwards as compared to continuation of SSRIs during pregnancy. We will study the effects on both mother and child with a pragmatic approach. Additionally, the study examines cost effectiveness. If non-inferiority of preventive cognitive therapy with guided tapering of SSRIs compared to intended continuation of SSRIs is demonstrated for the primary outcome, this may be the preferential strategy during pregnancy.

**Trial registration:**

Netherlands Trial Register (NTR): NTR4694; registration date: 16-jul-2014

## Background

Depressive disorder and anxiety disorders are the primary indications for the use of Selective Serotonin Reuptake Inhibitors (SSRIs). Worldwide, the SSRI prescription rate during pregnancy ranges from 6.2 % in the USA [1], to 3.7 % in the UK [2]. The actual Dutch nationwide estimated use of SSRIs during pregnancy is about two percent [3, 4]; while in the Rotterdam area this number is even as high as five percent [5]. Nonetheless, SSRI use during pregnancy is still controversial. On the one hand SSRIs may be toxic to the intrauterine developing child, while on the other hand, relapse of depression and/or anxiety during pregnancy poses risks for both mother and child [6].

The preventive effect of SSRIs for relapse of depression during pregnancy seems equivocal. One naturalistic study showed a significant increased risk of relapse in pregnant women who discontinued their medication compared to continuing medication (68 % vs. 26 %), while another naturalistic study showed no clear difference relapse rates of depression (16 % in total) between pregnant women continuing or discontinuing antidepressants [7, 8].

Pregnancy-related complications both exist for women using SSRIs during pregnancy and women with untreated depression/anxiety during pregnancy, posing a dilemma for the treating physician who considers SSRI withdrawal. For example, studies found significantly increased risks for preeclampsia among women who use SSRIs and increased risks for pregnancy-induced hypertension in women with depression/anxiety during pregnancy compared to healthy controls [9, 10].

Whether or not SSRIs are of direct influence on the newborn, both short- and long-term, is another unresolved issue. For example, a recent meta-analysis showed an increased risk for cardiovascular malformations (RR = 1.36) and septal heart defects (RR = 1.40) with use of SSRIs [11]. These findings were however not supported by a recent Nordic cohort study, which – after a sibling controlled analysis – found no substantial increase in prevalence of overall cardiac birth defects for any SSRI (OR = 0.92) [12]. Another example of evidence of a potential direct toxic effect is the association of SSRI use with persistent pulmonary hypertension (PPHN) of the neonate. A large cohort study from the Scandinavian national health registers showed a twofold-increased risk of PPHN with exposure later than gestational week 20 (OR = 2.1) [13]. However, this risk appeared more modest (OR = 1.51) in a large cohort study from 46 US states [14].

Several other effects of SSRIs during pregnancy have been described, such as a higher risk of poor neonatal adaptation (OR = 5.07), respiratory distress (OR = 2.20), tremors (OR = 7.89), preterm delivery and small for gestational age, lower birth weight and lower Apgar scores at 1 and 5 min after birth [15, 16]. Long-term effects on children are less often investigated. One systematic review found an adverse effect on children’s motor development but not on emotional or behavioural development [17]. Two large studies reported on the association between maternal SSRI use and childhood autism spectrum disorders, but found conflicting results [18, 19].

On the other hand, leaving depression or anxiety disorders untreated may be hazardous to the unborn child as well. At present, it is well known that children of women who suffered from anxiety or depression during pregnancy have an increased risk of adverse perinatal health outcomes, and behavioural, emotional, cognitive, and motor problems in early childhood [20, 21]. It is also shown that the infant cortisol stress response is altered if the mother suffered from depression during pregnancy [22]. One meta-analysis showed an association of depression during pregnancy with preterm birth and low birth weight [23]. Another more recent meta-analysis showed that depression during pregnancy is associated with premature delivery, but did not find associations with birth weight, neonatal intensive care unit admissions, preeclampsia, gestational age or Apgar scores [24].

Overall, in clinical practice and literature, pregnant women express a strong preference for non-pharmacologic treatment of depression over antidepressant medication [25]. Hence, cognitive behavioural therapy (CBT) could be a good alternative for SSRI use during pregnancy. According to a recent meta-analysis there is strong evidence that CBT interventions are effective for preventing depressive relapse during the perinatal period [26]. A recent follow-up study showed that preventive cognitive therapy (PCT) has long-term effects in preventing depressive relapse in patients with recurrent depression for over 5.5-10 years after the sessions ended [27, 28]. This preventive psychological strategy therefore seems promising in preventing depressive relapse, presumably also during pregnancy. Moreover, a recent study in the UK among non-pregnant patients showed that tapering antidepressants with therapy was as effective as continuation of antidepressants (Hazard Ratio 0.89) [29]. Nevertheless, further investigation is necessary to assess effectiveness of tapering antidepressants with added PCT during the perinatal period.

In conclusion, pregnant women and their clinicians face a dilemma, which is widely experienced in current practice [30]. At present, there are no suitable data available to guide evidence based decisions on SSRI continuation or discontinuation during pregnancy [31]. Both the National Institute for Health and Clinical Excellence in the United Kingdom (NICE) guideline [32], and American Psychiatric Association (APA) [33] therefore recommend to discuss both possibilities with women. The recently developed Dutch multidisciplinary guideline advises to continue SSRI use during pregnancy, and furthermore advises a hospital delivery and neonatal observation based on the increased risk and the severity of the (rare) condition of PPHN and prevalence (25–30 %) of children with neonatal abstinence after maternal SSRI use [34]. Nonetheless, the need of randomized trials was stressed. Indeed, existing studies are observational and therefore their results do not fully allow causal inference nor definite conclusions for practice.

### Trial objectives

In this randomized controlled trial (RCT), the effect of preventive cognitive therapy (PCT) with guided tapering of SSRIs in early pregnancy will be compared to continuation of SSRIs during pregnancy. We will study effects on both mother and child with a pragmatic approach. The expectation is that tapering of SSRIs with added PCT does not increase the risk of clinically relevant maternal relapse or recurrence^1^ of depression or onset of anxiety disorders during pregnancy up to three months postpartum in excess of [absolute] 15 % compared to continuation of SSRIs. If so, discontinuation is deemed non-inferior with regard to relapse/recurrence risk. Furthermore, we expect that tapering of SSRIs is better than continuation of SSRIs with respect to child development. Finally, but not unimportantly, we hypothesize that discontinuation will decrease total costs per woman and child on a 3 months and projected long term base, assuming no relevant effects of discontinuation on the mother and no effects on the child are found.

## Methods/Design

### Design & setting

**Fig. 1:**
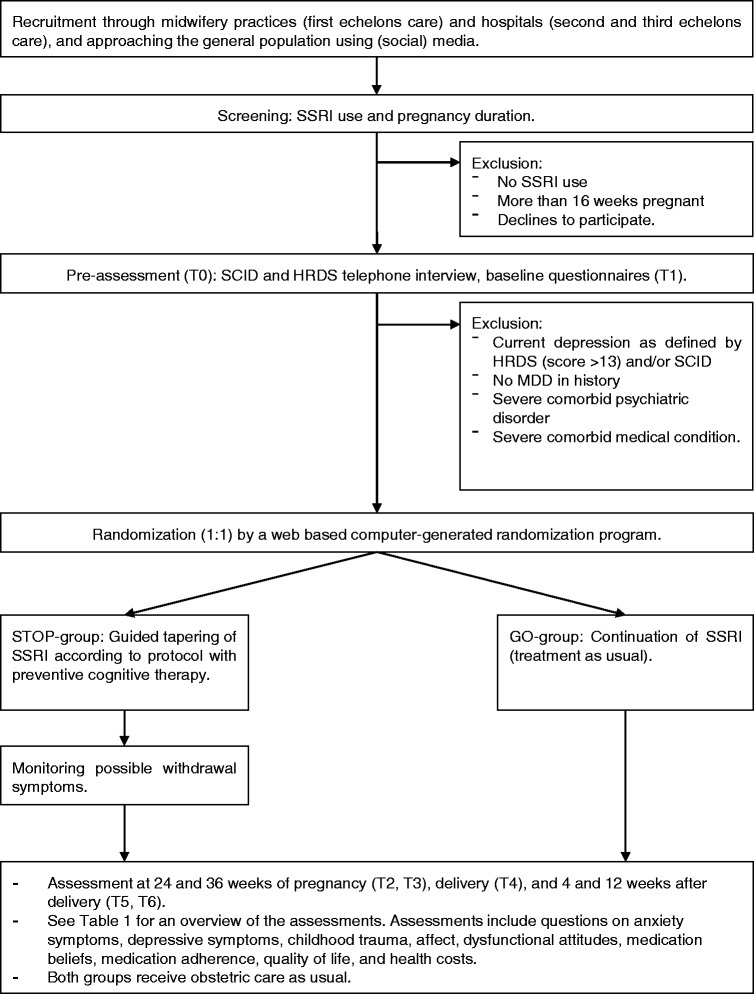
Study flow chart

The Stop or Go study is a pragmatic multi-centre randomized controlled non-inferiority trial (RCT) in obstetric care. Women will be recruited during their first prenatal visit in midwifery practices (first echelon care) and hospitals (second and third echelons care), or through advertisement in (social) media. After inclusion, women will be randomly allocated into two groups: STOP or GO. Both groups will receive regular assessments throughout their pregnancy and up to 3 months post-partum. Permission will be asked to contact the Centre of Childhood (CJG) at 24 months after delivery for information on the development of the child. Total duration of the study for participants will therefore be 30 months. In Fig. 1 an overview of the study design and main procedures is shown.

### Participants

Women who are less than 16 weeks pregnant and use a SSRI primarily for depressive disorder, and are currently at least in remission or recovered [35], are invited to participate in the trial. Exclusion criteria are multiple pregnancy, as these women have a markedly increased obstetric risk, thereby threatening the homogeneity of the study population and thus potentially complicate the statistical analysis, and insufficient proficiency in Dutch or English, since our intervention is not yet available in other languages. Also, women will be excluded with severe medical conditions, such as oncology-related conditions or conditions that need urgent medical interventions, which involve treatment decisions overriding research participation. Exclusion criteria related to mental health are: current mania or hypomania or a history of bipolar illness, suicidality and serious self harm, any psychotic disorder (current and previous), current alcohol or drug misuse, predominant anxiety disorders and personality disorders that require psychotherapeutic treatment for more than 2 sessions a month.

### Assessment of eligibility

After informed consent is obtained, a pre-assessment interview will be conducted, with the Structured Clinical Interview for DSM-disorders (SCID) [36] and the Hamilton Depression Rating Scale (HDRS) [37] to assess major DSM-IV Axis I psychiatric diagnoses and actual remission status and depressive symptoms respectively. Before randomization, study researchers will contact the medical professional who prescribed the SSRI medication to inform the professional about the study and discuss exclusion criteria as described above for study participation.

### Randomisation

Two hundred women will be randomized in a 1:1 allocation ratio to either the intervention arm (STOP) or the care as usual arm (GO). Randomization will be done with a web based computer-generated randomization schedule (a validated TENALEA Clinical Trial Data Management System; http://www.formsvision.com/) using permuted blocks of random size with a maximum of 16 and stratified for the number of previous depressive episodes (dichotomized). Based on a recent review [35], the participants are divided into groups of participants with 3 or less previous depressive episodes, versus 4 or more. Allocation of participants is concealed for study researchers.

### Interventions

#### Tapering SSRI

Women assigned to discontinuation of SSRIs will be referred to a psychiatrist trained in guiding tapering of SSRIs during pregnancy. They will plan and carry out SSRI discontinuation using an expert-based discontinuation protocol [38]. The aim is to taper the use of SSRIs within four weeks, depending on patient preferences and on drug characteristics (e.g., half-life in the body). There are no restrictions on the use of medication like sleeping pills, paracetamol, and mild tranquillizers. All co-medication will be monitored during the study period.

### Preventive cognitive therapy

Trained psychologists will provide preventive cognitive therapy in the discontinuation arm. This psychological intervention has proven to be effective in relapse prevention [27, 39–42]. The current manual was evaluated in previous studies [27, 38, 41, 43].

The intervention will be applied through VSee (http://www.vsee.com), a HIPAA-compliant telehealth app. Several studies demonstrated that psychological intervention as applied by telephone support is effective and there is some evidence that it might be effective to decrease postpartum depressive symptomatology [44–47]. Although not tested during pregnancy, there are indications that antenatal telephone or online therapy is effective and convenient [48].

The preventive psychological intervention consists of a minimum of eight weekly VSee sessions. These sessions are led by professional psychologists trained in cognitive behavioural therapy and may occur at any time of the day. The focus of the sessions is on identifying and teaching the participants to challenge dysfunctional beliefs, enhance recall of positive feelings and cognitions and a personal prevention plan is developed in which it is specified how the participant can prevent a depressive episode in the future. For each session the participant will receive some assignments of approximately 10 min per day. Treatment adherence will be monitored.

### Care as usual

Women assigned to continuation of SSRIs (GO) obtain usual care. They will be instructed to consult their doctor as they regularly do, in line with the pragmatic nature of the study. All the care that is provided will be monitored.

### Outcome measures

#### Mother

Primary outcome of this trial is (cumulative) incidence of relapse or recurrence of a depressive episode (as defined by the SCID-I [36]) during pregnancy and up to 12 weeks postpartum. The SCID-I is assessed at baseline (T0) and 12 weeks postpartum (T6). If – based on assessment with the HDRS at fixed time-points – relapse/recurrence is suspected, the SCID-I will be performed intermittently.

For registration of severity of depressive symptoms, the HDRS will telephonically be assessed additionally, at baseline (T0), at 36 weeks of gestation (T3), and 12 weeks postpartum (T6), and also intermittently, if necessary [37]. When the HDRS at any stage turn out above cut-off scores, the participant will be called one week after initial measurement. The HDRS will be repeated to confirm or reject elevated scores. An adjusted telephonic version of the everyday problem checklist (EPCL) and pregnancy related life events will be assessed during each telephonic measurement (T0, T2, T3, T5 and T6).

Women will be asked to fill in questionnaires during five occasions: baseline (T1), 24 and 36 weeks of gestation (T2 and T3), and 4 and 12 weeks postpartum (T5 and T6). The questionnaires differ in composition at the five measurement moments, as shown in Table 1. During these occasions, participants are variably asked to report on anxiety symptoms (Dutch version of the State Trait Anxiety Inventory STAI), short and long version [49, 50]), depressive symptoms (the Dutch version of the Edinburgh Postnatal Depressions Scale; EPDS [51]), childhood trauma (Childhood Trauma Questionnaire; CTQ [52]), affect (the International Short-Form of the Positive and Negative Affect Schedule; I-PANAS-SF [53]), dysfunctional attitudes (Dysfunctional Attitude Scale; DAS [54]), medication beliefs (Beliefs about Medicines Questionnaire; BMQ [55]), medication adherence (Medication Adherence Rating Scale; MARS [56]) and Quality of Life (EQ-5D-5L [57]). Socioeconomic position, ethnicity, smoking behaviour, alcohol use, family history and information on previous pregnancies and family size will be assessed using the Mind2Care questionnaire [58], a screen-and-advice instrument to detect mental health problems among pregnant women.

**Table 1:** Assessment per measurement moment

	Method	T0	T1	T2	T3	T4	T5	T6	T7
Clinical Diagnostic Interview (SCID-I)	Int	X		…	…	…	…	X	
Depressive symptoms (HDRS)	Int	X		…	X	…	…	X	
Peripartum depression (EPDS)	SR		X	X	X		X	X	
Anxiety (STAI)	SR		X	X	X		X	X	
Affect (I-PANAS-SF)	SR		X	X	X		X	X	
Attitudes (DAS)	SR		X	X	X				
Daily hassles	Int	X		X	X		X	X	
Life events	Int	X		X	X		X	X	
Sociodemographic & -economic factors (Mind2Care)	SR		X						
Substance use (Mind2Care)	SR		X	X	X		X	X	
Medication use	Int	X		X	X		X	X	
Medication adherence	SR		X						
Medication beliefs	SR		X						
Childhood trauma (CTQ)	SR			X					
Quality of Life (EQ-5D-5L)	SR		X	X	X		X	X	
Health care consumption (TIC-P)	SR		X		X		X	X	
Pregnancy related outcomes	CG					X			
Neural development (GM)	ME							X	
Child behaviour (CBCL)	SR								X
Cortisol (hair strands)	BM		X			X		X	
Buccal swab	BM		X					X	
Blood sample	BM							X	
Meconium (SSRI concentration)	BM					X			
Breast milk (SSRI concentration)	BM					X			

*Int* interview, *SR* self report, *CG* caregiver, *BM* biological materials, *T0* pre-assesment, *T1* baseline, *T2* 24 weeks of gestation, *T3* 36 weeks of gestation, *T4* delivery, *T5* 4 weeks postpartum, *T6* 12 weeks postpartum, *T7* 18 months postpartum

Health care cost data is registered using the TIC-P [59]. This instrument allows reliable recall over the past six months [60]. We will adapt scoring for ‘normal’ absenteeism and sickness leave for pregnant and recently delivered women. Care will be taken for secondary effects on child-care for other children (if present) in case of postpartum hospitalisation.

Using the Discontinuation Emergent Signs and Symptoms checklist (DESS) [61], the discontinuation group will be monitored by telephone weekly during tapering, to collect information about dosages and potential symptoms of withdrawal. Both groups will receive telephonic monitoring of medication use, including psychiatric co-medication, at 24 and 36 weeks of gestation (T2 and T3) and 4 and 12 weeks postpartum (T5 and T6).

Alongside the self-report measures, several sources of biological materials will be collected during the study. At baseline, immediately after delivery and 12 weeks postpartum (T1, T4 and T6) we will collect maternal hair strands to measure cortisol levels. Hair cortisol is a validated biomarker for long-term cortisol exposure and makes it possible to create a timeline of cortisol exposure during follow-up [62]. At baseline a maternal buccal swab will be collected in order to enable epigenetic and pharmacogenetic analysis. Maternal blood sampling will be performed 12 weeks postpartum (T6) to enable additional epigenetic and pharmacogenetic testing, but also for measurement of SSRI concentration and immunological factors.

### Health care professional

We will send a Case Report Form (CRF) to the participant’s obstetric caregiver, either a midwife or a gynaecologist, to request information about the pregnancy and delivery. Complications during pregnancy and delivery, such as hypertensive disorders or pregnancy, foetal growth retardation, preterm labour, induced labour and caesarean section will be registered as well as information about the neonate (e.g., Apgar scores, birth weight, congenital malformations and admission to paediatric ward).

### Child

At 12 weeks postpartum we will perform a General Movements (GM) assessment by taking video recordings at home [63]. This assessment method evaluates the function of the young nervous system.

GMs are spontaneous movements that are present from early foetal life onwards until the end of the first half-year of life. GMs are complex, occur frequently and last long enough to be observed properly. If the nervous system is impaired, GMs lose their complex and variable character and become monotonous and poor [64].

For mapping of the SSRI exposure of the newborn, samples of meconium and breast milk (if breastfeeding) will be collected. SSRI in meconium will be measured by a validated method according to LCH guidelines on LC-MS/MS [65]. If feasible, hair strands and a buccal swab of the newborn will be collected at 12 weeks after birth (T6).

Long-term follow-up includes the well-established, reliable and valid Child Behaviour Check List 1.5-5 years, including the Caregiver Teacher Report Form (C-TRF) and the Language Development Survey (LDS) at 18 months postpartum [66]. Also, permission will be asked to obtain routine data from Centres for Childhood (CJG) until 24 months (in particular on length gain, weight gain, normal development, and any information on abnormal behavioural development).

### Sample size

Sample size calculation is based on the main aim of this study, which is to demonstrate non-inferiority of preventive CT with guided discontinuation of SSRIs (STOP) compared to continuation (GO), with respect to relapse or recurrence of a depressive episode up to 3 months postnatal. We will use a non-inferiority margin (tolerance threshold, ‘delta’) of 15 %. This is based on the assumption that this excess relapse (taking into account the possibility of restoring SSRI treatment) is still in balance with the expected beneficial effects of discontinuation of SSRI for the remaining mothers. We also anticipate that this balance is acceptable for women.

With this non-inferiority margin, and the assumption that the overall absolute risk of relapse will be around 15 % [67], we need 178 women, given alpha .025, power 80 %, and a one-sided test. To account for some attrition, we aim to include 200 women in total. Given this sample size, we have sufficient power to demonstrate small to moderate effect sizes of .42 or over on continuous secondary outcomes. With respect to dichotomous secondary outcomes, we will be able to detect odds ratios of 1.5 or over when the base probability is .5.

### Statistical analysis

Analysis will primarily be carried out according to the intention-to-treat principle, i.e., the participants will be analysed according to their randomized allocation, regardless of the actual interventions received by the participant. Supplementary, we will perform analyses per protocol, i.e., according to actual SSRI use, irrespective of randomized arm.

The primary outcome, risk (cumulative incidence up to 3 months postnatal) of relapse of depression, will be compared between the randomized groups. Differences will be assessed statistically using a one-sided Chi-Square Test at a significance level of .025 and will be presented as a risk difference. The remainder of statistical tests will be performed two-sided at a significance level of .05.

Time to relapse will be compared between the randomized groups using survival analysis. Kaplan-Meier curves will be constructed and differences will be tested using the log-rank test. A Cox proportional hazard model will be used to calculate hazard ratios

Continuous outcomes, e.g., the General Movements scores at 3 months, will be compared between the groups using the unpaired *t*-test. Categorical secondary outcomes, e.g., obstetric complications, will be tested using Chi-Square Tests. For the continuous variables and categorical variables that are assessed more than twice, we will deploy linear mixed models and generalized linear mixed models respectively. These models use all available data (do not exclude persons with missing values) under the assumption of data being missing at random, and account for within-subject correlation over time. If despite randomization prognostically important factors differ between the groups, they will be adjusted for in supplemental analyses by including these factors in the pertaining regression models.

Subgroup analyses will be undertaken according to: Dutch/non-Dutch, nulliparous/multiparous, yes/no history depressive disorder and/or anxiety disorder, yes/no co-morbid anxiety symptoms or disorder. All effect parameters will be supplied with a 95 % confidence interval.

### Economic evaluation

In the present study we will also evaluate the outcome in the two study groups (Stop and Go) from a societal, economic perspective. It is therefore important to weigh cost savings for both groups against their clinical value. If relapse/recurrence incidence is within the predefined threshold (15 %), hence non-inferiority is confirmed; a straightforward cost minimization analysis will be executed focussing on cost savings. However, successful tapering of SSRIs will reduce SSRI use for years. Hence, with a sensitivity analysis on maternal effects and costs we will project cost estimations for 10 years. We expect that the upfront investment in PCT for women with previous psychiatric disorders will then be balanced by reduced SSRI use and less healthcare consumption. A previous RCT in a non-pregnant population demonstrated that a brief CT intervention is cost effective in remitted depressed individuals that stop antidepressants, compared to continuation of antidepressants [68].

If, however, relapse/recurrence incidence is higher than the predefined tolerance threshold, thus discontinuation is clinically inferior and rejected, a cost-effectiveness analysis will be executed as primary analysis, which estimates the costs avoided per additional relapse. This is the opposite of the extra costs per prevented relapse, if the starting point would have been no SSRI, and starting SSRI would be considered. Regardless the relapse outcome, we will conduct a cost utility analysis which estimates the impact of SSRI on the costs per Quality Adjusted Life Year (QALY), at least with a 3 month time horizon.

## Discussion

The use of SSRIs during pregnancy remains a clinical dilemma for both clinicians and patients. Given the increase of SSRI use among pregnant women and studies reporting conflicting results [7, 8, 11–14, 18, 19], there is dire need of randomized controlled trials investigating the use of SSRIs during pregnancy. This study will be the first to investigate the effect of preventive cognitive therapy with guided tapering of SSRIs from early pregnancy onwards as compared to continuation of SSRIs during pregnancy. Additionally, the study focuses on child outcomes and cost effectiveness.

Previous studies on relapse prevention showed promising results for tapering antidepressants with added relapse prevention [29]. Preventive cognitive therapy moreover showed promising long-term effects in non-pregnant women with a history of depression [27, 28]. Preventive cognitive therapy with guided tapering of antidepressants may therefore be a good alternative for SSRI use during pregnancy.

To our knowledge, no randomized controlled trials have been performed during pregnancy that investigated alternative treatment options versus SSRI use. This may be the result of the complex ethical situation of studies in pregnant women who are taking SSRIs and must be willing to either taper or continue SSRI use. Logistics of a nationwide randomized controlled trial are also difficult in a multidisciplinary setting. Although a multidisciplinary guideline exists, health care givers still have different views on best practice and therefore give different advices to their patients. This study will therefore be as pragmatic as possible, while still providing the intervention in a protocoled manner.

Results of this study will be published and will contribute to further development of (international) guidelines. The results will provide a first step in giving pregnant women an answer to the question whether it is better to stop or to continue the use of SSRIs during pregnancy.

## Ethics approval and consent to participate

The Medical Ethical Commission of the Erasmus Medical Center approved this study. Participants will sign informed consent form before participation.

## Abbreviations

BMQ: beliefs about medicines questionnaire
CBT: cognitive behavioural therapy
CJG: centres for childhood
CRF: case report form
CTQ: childhood trauma questionnaire
C-TRF: caregiver teacher report form
DAS: dysfunctional attitude scale
DESS: discontinuation emergent signs and symptoms checklist
EPCL: everyday problem checklist
EPDS: Edinburgh postnatal depressions scale
EQ-5D-5L: quality of life questionnaire
GM: general movements
HDRS: Hamilton depression rating scale
I-PANAS-SF: International short-form of the positive and negative affect schedule
LDS: language development survey
MARS: medication adherence rating scale
PCT: preventive cognitive therapy
PPHN: persistent pulmonary hypertension of the neonate
QALY: quality adjusted life year
RCT: randomized controlled trial
SCID-1: structured clinical interview for DSM-disorders axis 1
SSRI: selective serotonin reuptake inhibitors
STAI: State trait anxiety inventory

## Footnotes

1 Relapse is defined as the re-emergence of depressive symptoms during the remission phase, but before full recovery. Recurrence is defined as the onset of a new depression episode during the recovery phase or long remission phase.
